# Cancer treatment-related cardiovascular toxicity according to the ESC definition in patients receiving CAR T-cell therapy

**DOI:** 10.1186/s40959-026-00544-5

**Published:** 2026-07-30

**Authors:** Jakob Christoph Voran, Carolin Richard, Astrid Dempfle, Christiane Pott, Philipp Nakov, Guranda Chitadze, Natalie Schub, Catrin Meyer, Hatim Seoudy, Derk Frank, Claudia D Baldus, Oliver J Müller, David Baden

**Affiliations:** 1 Department of Internal Medicine III, Cardiology and Critical Care, University Hospital Schleswig-Holstein, Campus Kiel, Kiel, Germany; 2 partner site North, DZHK (German Centre for Cardiovascular Research), Kiel, Germany; 3 Department of Internal Medicine II, Haematology and Oncology, University Hospital Schleswig-Holstein, Campus Kiel, Arnold-Heller-Str. 3, Kiel, 24105 Germany; 4 University Cancer Centre Schleswig-Holstein (UCCSH), University Hospital Schleswig-Holstein, Kiel, Germany; 5 Institute of Medical Informatics and Statistics, Kiel University, University Hospital Schleswig-Holstein, Kiel, Germany; 6 Department of Internal Medicine V, Angiology, University Hospital Schleswig-Holstein, Campus Kiel, Arnold-Heller-Str. 3, Kiel, Germany

**Keywords:** CAR T-cell therapy, Cancer treatment related cardiovascular toxicity (CTR-CVT), Cancer therapy-related cardiac dysfunction (CTRCD)

## Abstract

**Background:**

Chimeric antigen receptor (CAR) T-cell therapy has substantially improved outcomes in refractory hematologic malignancies but may cause cardiovascular complications, particularly in the context of cytokine release syndrome (CRS). Real-world data on incidence, severity, and prognostic relevance of cancer therapy–related cardiovascular toxicity (CTR-CVT) defined by the 2022 ESC cardio-oncology guidelines remain in the context of CAR-T cell therapy limited.

**Methods:**

This retrospective single-center study includes 104 patients treated with CAR T-cells between 09/2019 and 02/2024 for acute lymphoblastic leukemia, non-Hodgkin lymphoma and multiple myeloma. The primary endpoint was new-onset CTR-CVT during hospital stay, defined as cancer therapy–related cardiac dysfunction (CTRCD), arrhythmia, myocardial infarction, cardiogenic shock, or cardiovascular death. Clinical characteristics, biomarkers, echocardiographic parameters, CRS/ICANS severity, and survival outcomes were analyzed.

**Results:**

Fifty-two patients (50%) met criteria for CTR-CVT, predominantly due to asymptomatic biomarker elevation. CTRCD occurred in 48.1%, whereas clinically significant events were rare: three patients developed symptomatic CTRCD, one experienced cardiogenic shock, and no myocardial infarctions or cardiovascular deaths were observed. Cardiovascular events occurred early and were associated with higher-grade CRS and ICANS, as well as elevated inflammatory markers. Reduced baseline left ventricular ejection fraction, elevated systolic pulmonary artery pressure, impaired performance status, and beta-blocker use were associated with increased CTR-CVT risk. Survival was numerically lower in patients with CTR-CVT but did not reach statistical significance.

**Conclusion:**

Although half of patients fulfilled ESC criteria for CTR-CVT, clinically relevant cardiac events after CAR T-cell therapy were uncommon. These findings suggest potential overclassification driven by biomarker elevations and underscore the importance of emphasizing clinical relevance when assessing cardiotoxicity in CAR T-cell recipients.

**Supplementary Information:**

The online version contains supplementary material available at 10.1186/s40959-026-00544-5.

## Introduction

Chimeric antigen receptor (CAR) T-cell therapy has transformed the treatment of hematologic malignancies, providing promising options for patients who previously had poor prognoses. CARs are synthetic fusion proteins combining a target-specific extracellular domain with intracellular signaling and costimulatory domains like CD28 or 4-1BB, redirecting autologous T cells to recognize and kill cells expressing specific antigens [1]. Since 2017, several CAR T-cell products targeting CD19 in acute lymphoblastic leukemia and lymphoma, as well as B-cell maturation antigen (BCMA) in multiple myeloma, have been approved by regulatory authorities. Given the unprecedented response rates observed in patients with previously limited therapeutic options, current research efforts are increasingly directed toward extending CAR T-cell therapy to solid tumors and nonmalignant conditions [2]. 

Cardiovascular and pulmonary complications occur in up to 10% of patients treated with CAR T-cell therapy, most commonly in the context of cytokine release syndrome (CRS) [3–5]. Early data from clinical trials likely underestimated these events, as patients with severe cardiac or pulmonary disease were often excluded. As CAR T-cell therapy is increasingly applied in real-world populations with significant comorbidities, data on potentially increasing toxicities is needed but sparse [5, 6]. While the management of CRS and immune effector cell–associated neurotoxicity syndrome (ICANS) has become part of routine clinical practice, cardiovascular events can pose immediate, potentially life-threatening risks. To assess the latter, the European Hematology Association (EHA), the European Society for Therapeutic Radiology and Oncology (ESTRO) and the International Cardio-Oncology Society recommended a cancer treatment related cardiovascular toxicity definition (CTR-CVT) and cancer therapy-related cardiac dysfunction (CTRCD) grading in their 2022 guideline on cardio-oncology [7]. Cancer therapy–related cardiovascular toxicity (CTR-CVT) is a composite term that includes all cardiovascular complications associated with anticancer therapy. Cancer therapy–related cardiac dysfunction (CTRCD) is one specific manifestation of CTR-CVT, describing treatment-related impairment of cardiac function and graded according to ESC-defined severity criteria. Recent studies suggest a low prevalence of cardiac toxicity, but substantial heterogeneity in patient populations and outcomes persists, and its prognostic significance remains unclear. This underscores the need for robust real-world data on incidence, severity, and outcomes using standardized criteria [8].

In this single-center analysis of patients treated with CAR T-cells between September 2019 and February 2024, we evaluated baseline cardiovascular risk, incidence and outcomes of CTR-CVT, and identified baseline and treatment-related predictors using detailed patient records.

## Methods

This retrospective cohort study examined the course of treatment and cardiovascular complications of all patients who received a CAR T-cell therapy at the Department of Hematology and Oncology at the University Medical Center Schleswig-Holstein Campus Kiel between 09/2019 and 03/2024. All patients received one of the six CAR T-cell products approved by the European Medicines Agency for refractory malignant lymphoid neoplasms according to the given indications: Axicabtagene Ciloleucel, Yescarta^®^; Tisagenlecleucel, Kymriah^®^; Brexucabtagene Autoleucel, Tecartus^®^; Isocabtagene Maraleucel, Breyanzi^®^; Idecabtagene Vicleucel, Abecma^®^ or Ciltacabtagen Autoleucel, Carvykti^®^.

Clinical data from before, during and after the CAR T-cell therapy were obtained from digital patient records including preexisting cardiovascular conditions (atrial fibrillation, heart failure, myocardial infarction, percutaneous coronary intervention, stroke or arterial vascular disease), cardiovascular risk factors, performance status, cancer stage, biomarkers, electrocardiogram and transthoracic echocardiogram (TTE) parameters, CRS and ICANS grade, the incidence of cardiovascular complications as well as the results from the last patient contact. The primary study endpoint was the development of cancer treatment related cardiovascular toxicity (CTR-CVT) according to the 2022 ESC Guidelines for cardio-oncology defined as new onset cancer therapy-related cardiac dysfunction (CTRCD), arrhythmia, myocardial infarction, cardiogenic shock or cardiovascular death during hospital stay ^7^. CTRCD levels were defined as follows according to the 2022 ESC guidelines (Supplement Table 1). To examine the left-ventricular ejection fractions (LVEF) changes for the diagnosis of CTRCD during CAR-T cell therapy, the results from TTE examinations before, during and after therapy, if available, were analysed. To identify a new rise in cardiac biomarkers, baseline levels of hsTnT and NT-proBNP were measured before CAR-T cell therapy and compared with peak values during the hospital stay. A new elevation was defined as a ≥ 50% increase from baseline in hsTnT or NT-proBNP. hsTnT > 14 ng/L (> 99th percentile) was considered elevated. NT-proBNP elevations used age-adjusted acute heart failure thresholds: >450 pg/mL (< 50 y), > 900 pg/mL (50–75 y), and > 1800 pg/mL (> 75 y) ^8^. Immunophenotyping was used to identify CD3, CD4, CD8, and CD19 positive cells prior to lymphodepletion.

After data collection, the cohort was divided into two groups: those who developed new-onset CTR-CVT during CAR-T cell therapy and those who did not. These groups were compared regarding their cardiovascular status prior to therapy, their clinical course during therapy, and findings from follow-up evaluations.

Categorical variables were reported as absolute numbers and percentages. Numerical variables were described using the median and interquartile range. To identify the influence of various variables on outcomes, logistic regression analysis was performed. The regression model accounted for the confounding factors age, sex, and pre-existing cardiac conditions. Overall survival (OS), progression-free survival (PFS) and relapse-free survival (RFS) were calculated from the date of CAR-T application. OS was defined as time to death from any cause, and PFS as time to relapse, progression, or death. RFS was defined as the time from CAR-T cell infusion until disease relapse or death from any cause, whichever occurs first, among patients who achieved a treatment response following CAR-T therapy.

Patients who died without prior relapse were excluded from RFS, and only those with documented disease control (partial or complete response) within the first six months of follow-up were included. Patients without response assessment were excluded. CAR-T product-specific event rates were calculated as the number of patients experiencing CTRCD divided by the total number of patients treated with each respective CAR-T product, and differences between products were assessed using a chi-square test. Laboratory and diagnostic continuous variables were compared using the Mann–Whitney U test, while categorical variables were analyzed using Fisher’s exact test or the chi-square test, as appropriate. Fisher’s exact test was used to assess whether the occurrence of CTR-CVT differed between CAR T-cell products. Missing data were visually assessed in R, and no clear systematic pattern of missingness was observed. Therefore, no imputation was performed, and analyses were conducted using available data, with the number of patients reported for each variable where applicable.

Data visualization and statistical analyses were performed using R (version 4.4.1) and IBM SPSS Statistics (version 29.0.2.0). A significance level of α = 0.05 was applied, with p-values ≤ 0.05 considered statistically significant.

The study was approved on November 1st, 2024, by the Ethics Committee of the Medical Faculty at Kiel University (D 610/24). As this was a retrospective analysis of pseudonymized routinely collected data, the ethics committee waived the requirement for individual patient consent.

## Results

During the observation period, 104 patients with elapsed or refractory (r/r) acute lymphoblastic leukemia, r/r non-Hodgkin lymphoma, and r/r multiple myeloma were treated. The median age was 63 years (IQR 53.2–70.8), and 26 patients (25.0%) were female. Median ECOG-Score was 1 (IQR0-1) and 73 patients (70.2%) were treated with an anthracycline based regimen prior to CAR T-cell therapy. The median length of stay in hospital was 21 days (IQR 18–26). Among the cohort, 42 patients had pre-existing cardiovascular diseases (e.g., atrial fibrillation, heart failure, myocardial infarction, history of percutaneous coronary intervention, prior cardiac surgery, stroke, or arterial vascular disease), 70 had known cardiovascular risk factors (hypertension, IDDM, diabetes, smoking, or dyslipidemia), and 30 patients had neither.

Of these 104 patients, 71 (68.2%) received CAR-T cell therapy for r/r non-Hodgkin lymphoma, including 53 with diffuse large B-cell lymphoma (50.9%), 9 with mantle cell lymphoma (8.7%), 5 with follicular lymphoma (4.8%), 2 with primary mediastinal B-cell lymphoma (1.9%), and 2 with high-grade B-cell lymphoma (1.9%). CAR-T therapy was also administered to 24 patients (23.1%) with r/r multiple myeloma and 9 patients (8.7%) with r/r B-cell acute lymphoblastic leukemia. A total of 90 patients received CAR-T products targeting CD19, including 41 with Axi-cel (Axicabtagene Ciloleucel, Yescarta^®^, 39.4%), 26 with Tisa-cel (Tisagenlecleucel, Kymriah^®^, 25%), 12 with Brexu-cel (Brexucabtagene Autoleucel, Tecartus^®^, 11.5%), and 1 with Liso-cel (Lisocabtagene Maraleucel, Breyanzi^®^, 1%). Additionally, 24 participants received BCMA-targeted CAR-T therapy, with 23 treated with Ide-cel (Idecabtagene Vicleucel, Abecma^®^, 22.1%) and 1 with Cilta-cel (Ciltacabtagene Autoleucel, Carvykti^®^, 1%, Supplement Table 2).

Among the 104 participants, 52 (50%) developed cancer therapy-related cardiovascular toxicity, constituting the CTR-CVT subgroup (Table 1). Of these, 50 patients (48.1%) experienced cancer therapy-related cardiac dysfunction (CTRCD). Clinically evident cardiovascular events were uncommon, as most CTRCD cases were mild and detected subclinically, including 43 asymptomatic (classified based on biomarker elevation, Supplement Table 3), 3 moderate asymptomatic, and 2 moderate symptomatic cases. Severe asymptomatic CTRCD and very severe symptomatic CTRCD occurred in 1 patient each, with the latter presenting with cardiogenic shock (Supplement Table 3). There was no statistically significant association between CAR-T product and CTR-CVT (*p* = 0.754). Atrial fibrillation occurred in 12 patients (11.5%) during CAR-T cell therapy; 4 of these had a prior history of atrial fibrillation, resulting in 8 cases (7.7%) classified as new-onset. Cardiovascular complications occurred at a mean of 4.6 ± 2.8 days after CAR-T cell administration. No myocardial infarctions or cardiovascular deaths were reported. Stratified by applied CAR-T product, CTRCD was found in 53.7% of patients treated with Axi-cel, 34.6% with Tisa-cel, 50.0% with Brexu-cel, 0% with Liso-cel, 56.5% with Ide-cel, and 0% with Cilta-cel. No CAR-T product was associated with a significantly higher rate of CTRCD (*p* = 0.43). The remaining 52 patients (50%) did not experience any CTR-CVT and were assigned to the second subgroup (“No CTR-CVT”).

**Table 1:** Number of patients with new onset of cancer treatment related cardiovascular toxicity

	*n*	Incidence rate (%)	95%-CI
CTR-CVT	52	50	40.4–59.6
thereof			
CTRCD	50	48.08	38.5–57.7
* CTRCD only*	*43*		
* CTRCD and Arrhythmia*	*6*		
* CTRCD and Cardiogenic Shock*	*1*		
Arrhythmia	8	7.7	2.6–12.8
Cardiogenic Shock	1	0.96	0-2.8
Myocardial infarction	0	0	0–0
Cardiovascular death	0	0	0–0

Incidence of CAR-T–related cardiovascular events out of 104 patients (CTR-CVT, n = 52). Events are shown overall and by type, with incidence rates and 95% confidence intervals

*Abbreviations:* *CTR-CVT* Cancer therapy-related cardiovascular toxicity, *CTRCD* Cancer therapy–related cardiac dysfunction

Analysis of the two subgroups showed that preexisting cardiac disease, cardiovascular risk factors, and abnormal ECG findings were more common in the CTR-CVT group, but differences were not statistically significant (Table 2). An ECOG performance status > 0 (OR 2.46, 95%CI 1.03–5.92, *p* = 0.016) and beta-blocker therapy (OR 2.81, 95%CI 1.04–7.58, *p* = 0.019) were linked to a significantly higher CTR-CVT risk. Regarding sex, BMI, prior anthracycline-based therapy or cancer stage, no significant differences between the groups were present.

**Table 2:** Patient characteristics stratified by CTR-CVT

	total cohort (*n* = 104)	CTR-CVT (*n* = 52)	no CTR-CVT (*n* = 52)	*p*-value
Median age, years (IQR)	62.5 (53.2–70.8)	65.1 (59.3–72.2)	57.8 (50.1–66.4)	0.041
Female, n (%)	26 (25)	13 (25)	13 (25)	1
Median BMI, kg/m^2^ (IQR)	25.7 (22.9–29.1)	25.9 (22.6–29.7)	25.5 (23.4–28.6)	0.706
Preexisting cardiac disease, n (%)	42 (40.4)	25 (48.1)	17 (32.7)	0.162
Cardiovascular risk, n (%)	70 (67.3)	38 (73.1)	32 (61.5)	0.296
ECOG > 0	69	40	29	**0.016**
Afib, n (%)	13 (12.5)	9 (8.7)	4 (3.8)	0.155
Previous PCI, n (%)	8 (7.7)	2 (1.9)	6 (5.8)	0.118
Dyslipidemia, n (%)	36 (34.6)	22 (21.2)	14 (13.5)	0.149
Beta blockers, n (%)	32 (30.8)	22 (21.2)	10 (9.6)	**0.019**
LVEF (*n* = 100, IQR)	58 (55–60)	55 (55–60)	60 (55–60)	**0.013**
LA-dilatation, n [%] (*n* = 97)	37 (38)	23 (23.7)	14 (14.4)	0.111
sPAP > 30mmHg, n [%] (*n* = 101)	28 (27.7)	20 (19.8)	8 (7.9)	**0.024**
QTc [ms] (*n* = 84)	430 (415–450)	438 (415–451)	428 (414–450)	0.447

Pre-CAR T-cell therapy evaluated characteristics of patients stratified by CTR-CVT outcome. P-values are exploratory and are not corrected for multiple testing. Age, BMI, LVEF and QTc were compared between groups using the Mann–Whitney U test, while categorical variables were analyzed using either Fisher’s exact test or the chi-square test, as appropriate depending on expected counts

*Abbreviations:* *Afib* Atrial fibrillation, *CTR-CVT* Cancer treatment related cardiovascular toxicity, *PCI* Percutaneous coronary intervention, *LVEF* Left ventricular ejection fraction, *LA* Left atrium, *sPAP* Systolic pulmonary artery pressure

Bold values indicate statistical significance (*p* < 0.05)

In the baseline TTE examination prior to CAR-T cell therapy, the median LVEF among the subgroup with CTR-CVT was slightly lower (55%) compared to the group without CTR-CVT (60%). An OR of 0.89 (95%CI: 0.81–0.98) in the regression model indicated a significant association between reduced LVEF and the occurrence of CTR-CVT (*p* = 0.013). Additionally, the data suggest a significantly increased risk of CTR-CVT in patients with an elevated systolic pulmonary arterial pressure (sPAP) above 30 mmHg with an OR of 2.91 (95%CI: 1.11–7.63) and a p-value of 0.01.

Patients developing CTR-CVT had lower baseline hemoglobin, and albumine levels, while inflammatory markers like Ferritin and IL-6 were found to be significantly higher in this group (Supplement Table 4). Patients with pre-existing cardiac disease or cardiovascular risk factors showed numerically higher, but non-significant, rates of CTR-CVT (pre-existing: *p* = 0.16; risk factors: *p* = 0.30). When considering either condition, the odds of CTR-CVT were higher but still not significant (OR 2.15, 95%CI 0.91–5.27; *p* = 0.13). Likewise, prior thoracic radiotherapy was not significantly associated with CTR-CVT (*p* = 0.21).

Additionally, lymphocyte populations including T and B cells, defined as CD3, CD4, CD8 positive, and CD19 positive, respectively, were measured in the sample of 73 participants during lymphocyte depletion (Supplement Table 5). Regression analysis did not identify statistically significant associations with cardiovascular complications.

Patients who developed CTR-CVT experienced higher rates of CRS and ICANS, accompanied by markedly elevated inflammatory markers, with peak ferritin over threefold and peak IL-6 more than twelvefold higher than in patients without cardiovascular complications. No correlation was observed between the administered CAR-T dose and the occurrence of CTR-CVT (Table 3). The occurrence of CRS of any grade differed significantly between CAR-T products (Fisher’s exact test, *p* < 0.001). Post-hoc pairwise analysis with Bonferroni correction showed that Axi-cel had a significantly higher CRS rate compared to Tisa-cel (97.6% vs. 61.5%, adjusted *p* = 0.002). Other pairwise comparisons were underpowered given the low number of patients in each group and did not reach statistical significance after correction.

**Table 3:** CAR-T-specific measurements stratified by CTR-CVT

	total cohort	CTR-CVT	no CTR-CVT	OR (95%-CI)	*p*-value
Peak CRP [mg/l] (*n* = 103)	126 (64.6-187.5)	140.5 (87.1-199.3)	115 (53.5–184)	-	0.029
Peak Ferritin [ng/ml] (*n* = 70)	1118 (384–2200)	1877 (703–6822)	550 (319–1308)	-	**< 0.001**
Peak IL-6 [ng/l] (*n* = 103)	402 (103–4147)	2068.5 (411.5-7152.5)	166 (53.5-396.5)	-	**< 0.001**
CRS > 1	44	31	13	4.37 (1.87–10.2)	**0.001**
ICANS > 0	39	26	13	2.8 (1.19–6.58)	**0.018**
Max. CART [/µl] (*n* = 44)	141 (43–303)	143 (54–282)	124 (41–305)	-	0.817

CAR-T specific outcomes and peak inflammatory markers stratified by CTR-CVT. P-values are exploratory and are not corrected for multiple testing. Peak values were compared by Mann-Whitney-U-Test

*Abbreviations:* *CRS* Cytokine release syndrome, *CTR-CVT* Cancer treatment related cardiovascular toxicity, *ICANS* Immune Effector Cell-Associated Neurotoxicity Syndrome, *Max. CART* highest CAR T-cell concentration measured

Bold values indicate statistical significance (*p* < 0.05)

## Survival

**Fig. 1:**
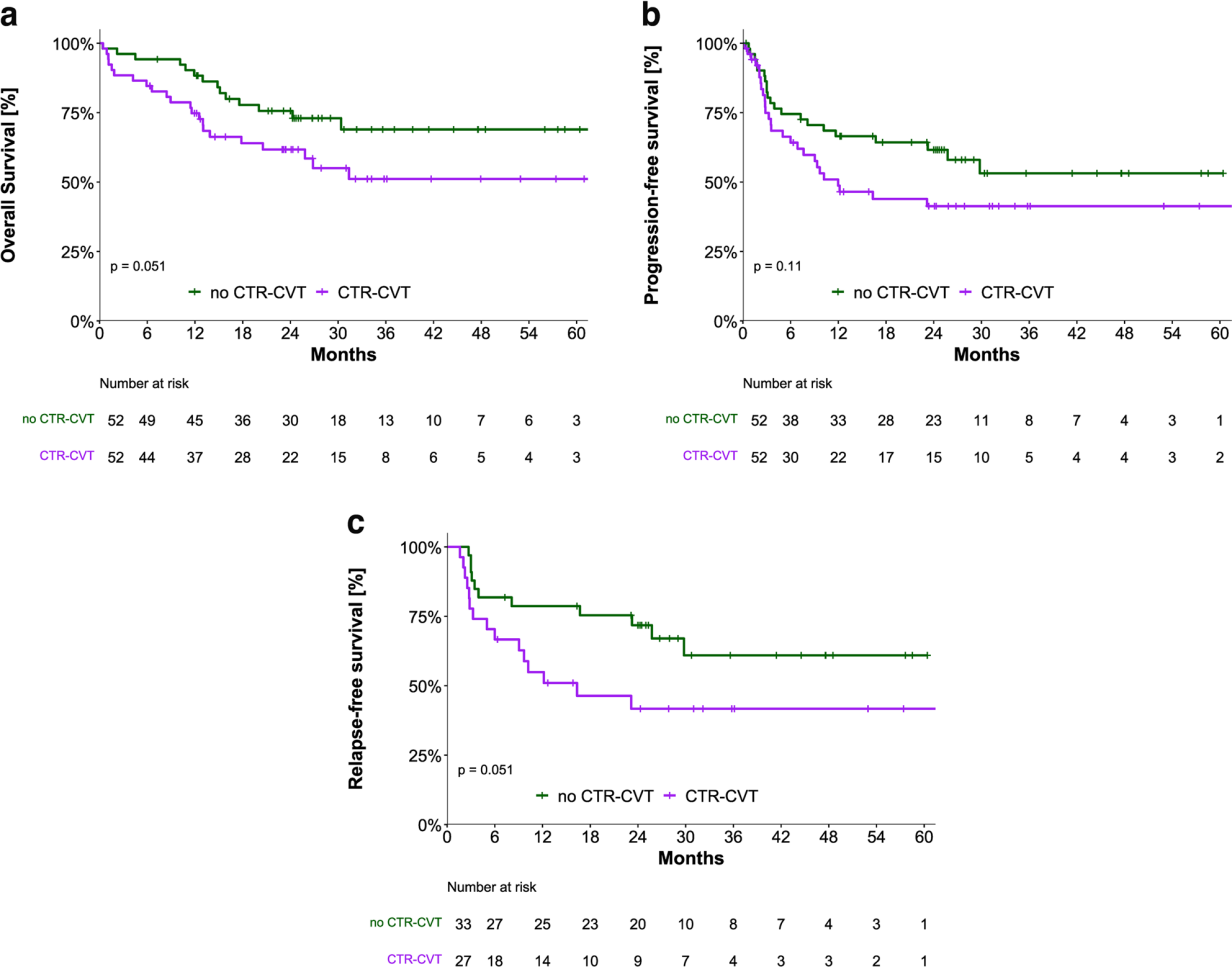
Overall (**a**), progression-free (**b**) and relapse-free (**c**) survival of CAR T-cell patients stratified by CTR-CVT. Patients who have not experienced CTR-CVT are depicted in green while CTR-CVT patients are plotted in purple. Survival was calculated from the application of CAR T-cells. Abbreviation: CTR-CVT = cancer treatment related cardiovascular toxicity

To investigate the relevance of CTR-CVT in terms of survival, we analyzed the OS of patients stratified by CTR-CVT (Fig. 1a). During the study period, 37 participants deceased. The reported causes of death were cancer progression, infection or sepsis due to underlying immunodeficiency, with no cases attributed to primary cardiovascular causes. Median follow-up time was 29.0 months (95%CI 23.4–44.5). Although we observed a tendency towards reduced survival in patients with CTR-CVT, significance was not reached (3y OS 51.1% (95%CI 37.5–69.6%) vs. 68.9% (95%CI 56.1–84.8%); *p* = 0.051). Likewise, progression-free and relapse-free survival showed a trend towards impaired outcomes in CTR-CVT patients without reaching statistical significance (Fig. 1b–c).

**Fig. 2:**
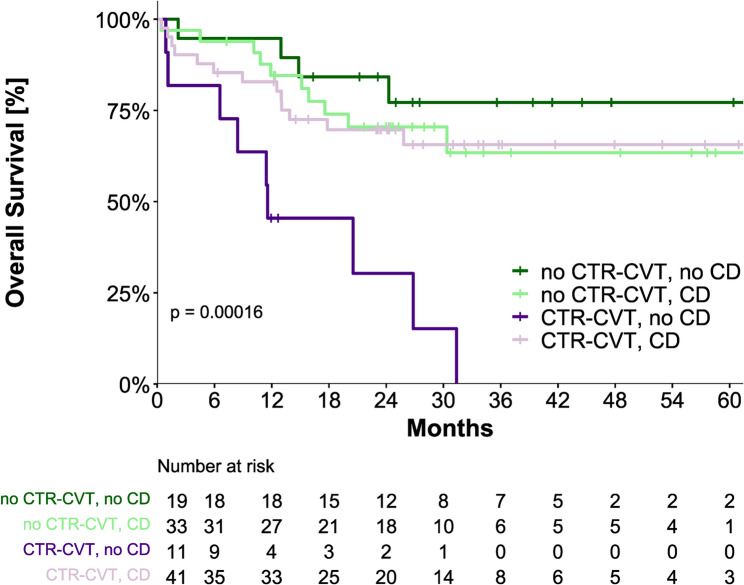
Overall survival of CAR T cell therapy patients stratified by CTR CVT status and baseline cardiac conditions or risk factors. CD = pre existing cardiac disease or cardiovascular risk factors. *P* value from log rank test

Interestingly, in 11 patients with CTR-CVT but no prior cardiac disease or risk factors a significantly worse survival compared to those without CTR-CVT or with CTR-CVT plus existing cardiac conditions was observed (median OS 11.57 (95%CI 8.43-NA) vs. not reached); Fig. 2). Apart from a slightly lower BMI (23.6 vs. 26.6 kg/m², *p* = 0.003), lower platelet counts (87.6/nl vs. 164.9/nl, *p* < 0.001) and increased peak ferritin (7643 µg/l vs. 1645 µg/l, *p* < 0.001), univariate analysis did not reveal other baseline, disease, treatment, or toxicity variables that could explain this difference. However, the limited sample size could lead to observational bias.

## Discussion

This European single-center study found a low incidence of clinically significant cardiovascular events after CAR-T therapy. Although 50% of patients developed CTR-CVT, this was primarily driven by CTRCD cases, most of which were asymptomatic and identified solely by elevated cardiac biomarkers. One cardiogenic shock occurred, and no cardiovascular deaths were observed, suggesting CAR-T therapy carries a low risk of severe cardiac complications.

This is in line with a recent meta-analysis reporting a low prevalence of serious adverse cardiac events in CAR T-cell recipients [9]. Similar to our analysis, arrhythmias and ventricular dysfunction were the most commonly observed events, whereas severe cardiac complications like myocardial infarction or cardiovascular death were rare. Accordingly, we also observed only a few severe cardiac events in an analysis of all CAR-T cell treatments in Germany published recently [10].

Patients with CTR-CVT more frequently had reduced performance status, beta-blocker treatment, decreased LVEF, impaired blood counts, and elevated inflammatory markers prior to CAR-T therapy, suggesting that frail patients or those with greater comorbidity burden may be more susceptible to cardiovascular complications during intensive therapies. This aligns with prior observations in patients undergoing autologous or allogeneic hematopoietic stem cell transplantation [11]. However, CTR-CVT was also associated with higher rates of CRS and ICANS. While causality cannot be established, the strong link between CRS and CTR-CVT indicates that systemic inflammatory responses induced by CAR-T therapy might cause microvascular obstruction, distributive shock, ventricular dysfunction, and myocardial ischemia and thereby directly contribute to cardiac toxicity. Notably, age and frailty do not appear to majorly influence CRS, supporting the idea that CRS and frailty are independent key drivers of CTR-CVT [12]. Due to the small cohort, we could not link CTR-CVT to a specific CAR-T product, although literature suggests some products may carry higher complication risks [13]. But consistent with previous reports, we observed higher CRS rates in patients treated with Axi-cel compared to those receiving Tisa-cel, which may increase the risk of cardiovascular complications and further raise treatment costs in general [14].

CD3-, CD4-, CD8-, and CD19-positive cells were quantified prior to lymphodepletion. We hypothesized that higher numbers of T cells (CD3+, CD4+, CD8+), increased CD19-positive target cells, or an altered CD4/CD8 ratio could amplify cytokine release during CAR T-cell therapy, causing systemic inflammation, cardiac stress, and an increased risk of CAR T-cell-related cardiovascular toxicity (CTR-CVT). Supporting this, higher CD3 + counts at leukapheresis are associated with elevated peripheral lymphocyte counts on day 7 post-infusion [15], and stronger CAR-T expansion has been linked to a higher risk of CRS. Additionally, the CD4/CD8 ratio of infused CD19-CAR T-cells is prognostic for both efficacy and toxicity, with CD4 + CAR-T cells identified as primary drivers of CRS [16–18]. In our cohort, we observed no statistically significant differences between both groups. Whether this reflects limited sample size or more complex biology remains unclear and warrants further studies.

Notably, the ESC 2022 cardio-oncology guidelines classify nearly half of CAR-T patients in our cohort as having CTR-CVT, despite a very low incidence of clinically diagnosed symptomatic cardiotoxicity. While the guidelines acknowledge that CRS commonly leads to elevated cardiac enzymes, this rate appears disproportionately high. A large study cited in these guidelines of 2,657 patients potentially supports the need for high vigilance, reporting a 30.9% fatality rate among 546 patients with cardiovascular and pulmonary adverse events; however, many deaths were due to respiratory or pleural complications and venous thromboembolism rather than direct cardiac toxicity [19]. Moreover, this analysis was limited to Axi-cel and Tisa-cel. Data from the CARDIOTOX registry showed that mild, objectively impaired cardiac function is common during cancer therapy, whereas severe cardiotoxicity and adverse prognosis are rare [20]. Similarly, Mecinaj et al. questioned the CTRCD definition after observing frequent mild, asymptomatic CTRCD without clinical consequences in patients receiving adjuvant breast cancer therapy [21]. Elevated cardiac enzymes do not necessarily reflect persistent injury or predict future complications and, in the case of BNP, may result from altered distributive hemodynamics and volume shifts during CRS. These findings suggest possible overclassification in the ESC guidelines and highlight the importance of emphasizing clinical symptoms and relevance, particularly in CAR T-cell–treated patients.

Patients with CTR-CVT showed numerically reduced survival, though differences were not statistically significant. RFS and PFS showed a similar pattern, indicating that poorer cancer-related outcomes and diminished treatment responses likely underly the reduced OS observed in this cohort. Furthermore, these patients exhibited higher rates of CRS and ICANS, more frequent performance impairment, and abnormal blood counts, indicating generally higher complication rates while having reduced fitness. Thereby, these observations do not establish a causal link between CTR-CVT and impaired survival.

Interestingly, 11 patients with CTR-CVT in the absence of prior cardiac disease or established cardiovascular risk factors showed significantly inferior survival compared with both patients without CTR-CVT and those with CTR-CVT plus pre-existing cardiac conditions. CTR-CVT in patients without known risk factors may represent a more acute or treatment-related toxicity that is less anticipated and potentially recognized later, whereas patients with pre-existing cardiac disease might benefit from closer monitoring and earlier intervention. As no relevant baseline or treatment differences were identified aside from a slightly lower BMI, unmeasured confounding or residual bias may explain the association (Supplement Table 6). Given the small sample size, the finding should be interpreted cautiously, hypothesis-generating and confirmed in larger studies.

As a retrospective single-center study, our analysis has inherent limitations, including a small sample size, lack of systematic follow-up, and potential surveillance, selection or misclassification biases.

Overall, cardiovascular complications during CAR-T therapy are rare and are primarily associated with CRS or ICANS. Cardiac events suggest monitoring should focus on ejection fraction declines and supraventricular arrhythmias, especially in frail patients or those with preexisting heart disease. Prospective studies with systematic cardiac assessment are needed to confirm these findings and detect rare events.

## Supplementary Information

Supplementary Material 1.

## Data Availability

All data supporting the results of this study are available upon reasonable request from the corresponding authors.

## References

[1] Brudno JN, Maus MV, Hinrichs CS. CAR T Cells and T-Cell Therapies for Cancer: A Translational Science Review. JAMA. 2024;332:1924–35. 10.1001/jama.2024.19462. 39495525 10.1001/jama.2024.19462 PMC11808657

[2] Rampotas A, Richter J, Isenberg D, Roddie C. CAR-T cell therapy embarks on autoimmune disease. Bone Marrow Transpl. 2025;60:6–9. 10.1038/s41409-024-02429-6. 10.1038/s41409-024-02429-6 PMC11726457 39379698

[3] Gill J. Cardiovascular Toxicities with Chimeric Antigen Receptor T-cell Therapy. Curr Cardiol Rev. 2023;19:e230622206353. 10.2174/1573403X18666220623152350. 35747980 10.2174/1573403X18666220623152350 PMC10201875

[4] Ganatra S, Dani SS, Yang EH, Zaha VG, Nohria A. Cardiotoxicity of T-Cell Antineoplastic Therapies: JACC: CardioOncology Primer. JACC CardioOncol. 2022;4:616–23. 10.1016/j.jaccao.2022.07.014. 36636447 10.1016/j.jaccao.2022.07.014 PMC9830211

[5] Jacobson CA, Munoz J, Sun F, Kanters S, Limbrick-Oldfield EH, Spooner C, Mignone K, Ayuk F, Sanderson R, Whitmore J, Wang Y, Xu H, Dickinson M. Real-World Outcomes with Chimeric Antigen Receptor T Cell Therapies in Large B Cell Lymphoma: A Systematic Review and Meta-Analysis. Transpl Cell Ther. 2024;30:77e1. 77.e15. 10.1016/j.jtct.2023.10.017 37890589

[6] Deschênes-Simard X, Bromberg M, Devlin SM, Gonen M, Beyar-Katz O, Ip A, Marcus R, Avigdor A, Ballweg A, Rabinovich E, Alhomoud M, Rivas Delgado A, Corona De Lapuerta M, De Luna A, Palomba ML, Shah GL, Lin R, Boardman AP, Falchi L, Lue J, Salles G, Perales M-A, Shouval R, Dahi PB, Scordo M. Comparative real-world outcomes of CD19-directed CAR T-cell therapies in large B-cell lymphoma. Blood Adv. 2025;9:5571–84. 10.1182/bloodadvances.2025016778. 40815804 10.1182/bloodadvances.2025016778 PMC12615284

[7] Lyon AR, López-Fernández T, Couch LS, Asteggiano R, Aznar MC, Bergler-Klein J, Boriani G, Cardinale D, Cordoba R, Cosyns B, Cutter DJ, de Azambuja E, de Boer RA, Dent SF, Farmakis D, Gevaert SA, Gorog DA, Herrmann J, Lenihan D, Moslehi J, Moura B, Salinger SS, Stephens R, Suter TM, Szmit S, Tamargo J, Thavendiranathan P, Tocchetti CG, van der Meer P, van der Pal HJH. 2022 ESC Guidelines on cardio-oncology developed in collaboration with the European Hematology Association (EHA), the European Society for Therapeutic Radiology and Oncology (ESTRO) and the International Cardio-Oncology Society (IC-OS). Eur Heart J. 2022;43:4229–361. 10.1093/eurheartj/ehac244. 36017568 10.1093/eurheartj/ehac244

[8] Korell F, Entenmann L, Romann S, Giannitsis E, Schmitt A, Müller-Tidow C, Frey N, Dreger P, Schmitt M, Lehmann LH. Evaluation of all-cause mortality and cardiovascular safety in patients receiving chimeric antigen receptor T cell therapy: a prospective cohort study. EClinicalMedicine. 2024;69:102504. 10.1016/j.eclinm.2024.102504. 38544797 10.1016/j.eclinm.2024.102504 PMC10965403

[9] Koeckerling D, Reddy RK, Barker J, Eichhorn C, Divall P, Howard JP, Korell F, Schmitt M, Dreger P, Frey N, Lehmann LH. Cardiovascular Events After Chimeric Antigen Receptor T-Cell Therapy for Advanced Hematologic Malignant Neoplasms: A Meta-Analysis. JAMA Netw Open. 2024;7:e2437222. 10.1001/jamanetworkopen.2024.37222. 39374017 10.1001/jamanetworkopen.2024.37222 PMC11459246

[10] Voran JC, Fransecky L, Seoudy H, Hecht M, Müller O, Berning P, Baden C, Versteegen T, Baldus CD, Stölzel F, Frank D, Baden D. *Low cardiovascular event rates in patients treated with CAR T-cells: Real-world outcomes from two independent cohorts*, 2026. 10.1093/eschf/xvag132 42102267

[11] Vasbinder A, Hoeger CW, Catalan T, Anderson E, Chu C, Kotzin M, Xie J, Kaakati R, Berlin HP, Shadid H, Perry D, Pan M, Takiar R, Padalia K, Mills J, Meloche C, Bardwell A, Rochlen M, Blakely P, Leja M, Banerjee M, Riwes M, Magenau J, Anand S, Ghosh M, Pawarode A, Yanik G, Nathan S, Maciejewski J, Okwuosa T, Hayek SS. Cardiovascular Events After Hematopoietic Stem Cell Transplant: Incidence and Risk Factors. JACC CardioOncol. 2023;5:821–32. 10.1016/j.jaccao.2023.07.007. 38205002 10.1016/j.jaccao.2023.07.007 PMC10774793

[12] Davis JA, Dima D, Ahmed N, DeJarnette S, McGuirk J, Jia X, Raza S, Khouri J, Valent J, Anwer F, Abdallah A-O, Hashmi H. Impact of Frailty on Outcomes after Chimeric Antigen Receptor T Cell Therapy for Patients with Relapsed/Refractory Multiple Myeloma. Transpl Cell Ther. 2024;30:298–305. 10.1016/j.jtct.2023.12.015. 10.1016/j.jtct.2023.12.015 38142943

[13] Mahadevia H, Modi K, Shah R, Modi S, Vojjala N, Khumri T, Vodnala D, Cossor F. Incidence of cytokine release syndrome (CRS), immune effector cell-associated neurotoxicity syndrome (ICANS), infections and cardiovascular events across different chimeric antigen receptor (CAR) T-cell therapy products in large B-cell lymphoma (DLBCL): A nationwide analysis. Blood. 2025;146:8012. 10.1182/blood-2025-8012.

[14] Kwon M, Iacoboni G, Reguera JL, Corral LL, Morales RH, Ortiz-Maldonado V, Guerreiro M, Caballero AC, Domínguez MLG, Pina JMS, Mussetti A, Sancho JM, Bastos-Oreiro M, Catala E, Delgado J, Henriquez HL, Sanz J, Calbacho M, Bailén R, Carpio C, Ribera JM, Sureda A, Briones J, Hernandez-Boluda JC, Cebrián NM, Martin JLD, Martín A, Barba P. Axicabtagene ciloleucel compared to tisagenlecleucel for the treatment of aggressive B-cell lymphoma. Haematologica. 2023;108:110–21. 10.3324/haematol.2022.280805. 35770532 10.3324/haematol.2022.280805 PMC9827173

[15] Wada F, Jo T, Arai Y, Kitawaki T, Mizumoto C, Kanda J, Nishikori M, Yamashita K, Nagao M, Takaori-Kondo A. T-cell counts in peripheral blood at leukapheresis predict responses to subsequent CAR-T cell therapy. Sci Rep. 2022;12:18696. 10.1038/s41598-022-23589-9. 36333521 10.1038/s41598-022-23589-9 PMC9636390

[16] Galli E, Bellesi S, Pansini I, Di Cesare G, Iacovelli C, Malafronte R, Maiolo E, Chiusolo P, Sica S, Sorà F, Hohaus S. The CD4/CD8 ratio of infused CD19-CAR-T is a prognostic factor for efficacy and toxicity. Br J Haematol. 2023;203:564–70. 10.1111/bjh.19117. 37789569 10.1111/bjh.19117

[17] Boulch M, Cazaux M, Cuffel A, Ruggiu M, Allain V, Corre B, Loe-Mie Y, Hosten B, Cisternino S, Auvity S, Thieblemont C, Caillat-Zucman S, Bousso P. A major role for CD4 + T cells in driving cytokine release syndrome during CAR T cell therapy. Cell Rep Med. 2023;4:101161. 10.1016/j.xcrm.2023.101161. 37595589 10.1016/j.xcrm.2023.101161 PMC10518592

[18] Lionel AC, Neelapu SS. CAR T-cell expansion: harmful or helpful? Blood Adv. 2024;8:3311–3. 10.1182/bloodadvances.2024013146. 38916899 10.1182/bloodadvances.2024013146 PMC11258617

[19] Goldman A, Maor E, Bomze D, Liu JE, Herrmann J, Fein J, Steingart RM, Mahmood SS, Schaffer WL, Perales M-A, Shouval R. Adverse Cardiovascular and Pulmonary Events Associated With Chimeric Antigen Receptor T-Cell Therapy. J Am Coll Cardiol. 2021;78:1800–13. 10.1016/j.jacc.2021.08.044. 34711339 10.1016/j.jacc.2021.08.044 PMC8562317

[20] López-Sendón J, Álvarez-Ortega C, Zamora Auñon P, Buño Soto A, Lyon AR, Farmakis D, Cardinale D, Canales Albendea M, Feliu Batlle J, Rodríguez Rodríguez I, Rodríguez Fraga O, Albaladejo A, Mediavilla G, González-Juanatey JR, Martínez Monzonis A, Gómez Prieto P, González-Costello J, Serrano Antolín JM, Cadenas Chamorro R, López Fernández T. Classification, prevalence, and outcomes of anticancer therapy-induced cardiotoxicity: the CARDIOTOX registry. Eur Heart J. 2020;41:1720–9. 10.1093/eurheartj/ehaa006. 32016393 10.1093/eurheartj/ehaa006

[21] Mecinaj A, Gulati G, Ree AH, Gravdehaug B, Røsjø H, Steine K, Wisløff T, Geisler J, Omland T, Heck SL. Impact of the ESC Cardio-Oncology Guidelines Biomarker Criteria on Incidence of Cancer Therapy-Related Cardiac Dysfunction. JACC CardioOncol. 2024;6:83–95. 10.1016/j.jaccao.2023.10.008. 38510299 10.1016/j.jaccao.2023.10.008 PMC10950440

