# Supplementary material for: Cancer treatment-related cardiovascular toxicity according to the ESC definition in patients receiving CAR T-cell therapy
Source: Cardiooncology. 2026 Jul 30;12:103. doi: 10.1186/s40959-026-00544-5 (PMC13425954; doi:10.1186/s40959-026-00544-5)
Supplement: Supplementary file 1 — Supplementary Material 1. [file 40959_2026_544_MOESM1_ESM.docx]

Cancer treatment related cardiovascular toxicity according to ESC definition under CAR T-cell treatment - Supplement

Jakob C Voran^*1,2^, Carolin Richard^*1^, Astrid Dempfle^5^, Christiane Pott^3,4^, Philipp Nakov^3,4^, Guranda Chitadze^3,4^, Natalie Schub^3,4^, Catrin Meyer^3,4^, Hatim Seoudy^1,2^, Derk Frank^1,2^, Claudia D Baldus^3,4^, Oliver J Müller^*#2,6^, David Baden^*#3,4^

***1*** *Department of Internal Medicine III, Cardiology and Critical Care, University Hospital Schleswig-Holstein, Campus Kiel, Kiel, Germany.*

***2*** *DZHK (German Centre for Cardiovascular Research), partner site North*

***3*** *Department of Internal Medicine II, Haematology and Oncology, University Hospital Schleswig-Holstein, Kiel.*

***4*** *University Cancer Centre Schleswig-Holstein (UCCSH), University Hospital Schleswig-Holstein, Kiel.*

***5*** *Institute of Medical Informatics and Statistics, Kiel University, University Hospital Schleswig-Holstein, Kiel, Germany.*

***6*** *Department of Internal Medicine V, Angiology, University Hospital Schleswig-Holstein, Campus Kiel, Kiel, Germany.*

**Supplement Table 1: Definitions and grading of cancer therapy–related cardiac dysfunction (CTRCD)**

| **Symptomatic CTRCD** | Very severe | HF requiring inotropic support, mechanical circulatory support, or consideration of transplantation |
| --- | --- | --- |
|  | severe | HF hospitalization |
|  | moderate | Need for outpatient intensification of diuretic and HF therapy |
|  | mild | Mild HF symptoms, no intensification of therapy required |
| **Asymptomatic CTRCD** | severe | New LVEF reduction to <40% |
|  | moderate | New LVEF reduction by ≥10 percentage points to an LVEF of 40–49% OR New LVEF reduction by <10 percentage points to an LVEF of 40–49% AND either new relative decline in GLS by >15% from baseline OR new rise in cardiac biomarkers |
|  | mild | LVEF ≥ 50% AND new relative decline in GLS by >15% from baseline AND/OR new rise in cardiac  biomarkers |

Supplement Table 1: Grading of cancer therapy–related cardiac dysfunction (CTRCD). CTRCD severity was classified based on symptoms, therapy requirements, and changes in LVEF, GLS, or cardiac biomarkers. Symptomatic cases were graded from mild to very severe, while asymptomatic cases were defined by new functional or biomarker changes.

*Abbreviations: CTRCD: cancer therapy–related cardiac dysfunction; HF: heart failure; LVEF; left ventricular ejection fraction; GLS: global longitudinal strain*

**Supplement Table 2: Patient numbers by CAR-T product and indication**

| **CAR-T product** | **Indication** | **n** |
| --- | --- | --- |
| Axicabtagene Ciloleucel (n=41) | DLBCL HGBCL PMBCL | 37 2 2 |
| Tisagenlecleucel (n=26) | DLBCL  FL  B-ALL | 15  5  6 |
| Brexucabtagene Autoleucel (n=12) | MCL B-ALL | 9 3 |
| Lisocabtagene Maraleucel (n=1) | DLBCL | 1 |
| Idecabtagene Vicleucel (n=23) | MM | 23 |
| Ciltacabtagene Autoleucel (n=1) | MM | 1 |

Supplement Table 2: Patient numbers per administered CAR-T product by indication (Axicabtagene Ciloleucel, Yescarta^®^; Tisagenlecleucel, Kymriah^®^; Brexucabtagene Autoleucel, Tecartus^®^; isocabtagene Maraleucel, Breyanzi^®^; Idecabtagene Vicleucel, Abecma^®^ or Ciltacabtagen Autoleucel, Carvykti^®^).

*Abbreviations: DLBCL: Diffuse Large B-Cell Lymphoma; HGBCL: High-Grade B-Cell Lymphoma; PMBCL: Primary Mediastinal B-Cell Lymphoma; FL: Follicular Lymphoma; B-ALL: B-Cell Acute Lymphoblastic Leukemia; MCL: Mantle Cell Lymphoma; MM: Multiple Myeloma*

**Supplement Table 3: Number of patients with new onset of cancer therapy-related cardiac dysfunction**

| **Type of CTRCD** | **n** | **Incidence rate (%)** | **95%-CI** |
| --- | --- | --- | --- |
| **Very severe symptomatic** | 1 | 0.96 | 0-2.8 |
| **Severe symptomatic** | 0 | 0 | 0-0 |
| **Moderate symptomatic** | 2 | 1.9 | 0-4.5 |
| **Mild symptomatic** | 0 | 0 | 0-0 |
| **Severe asymptomatic** | 1 | 0.96 | 0-2.8 |
| **Moderate asymptomatic** | 3 | 2.9 | 0-6.1 |
| **Mild asymptomatic**  *Due to hs-TNT elevation only* *Due to NT-proBNP elevation only* *Due to elevation of both* | 43  *2* *30* *11* | 41.35 | 31.89-50.81 |

Supplement Table 3: Incidence and severity of cancer therapy–related cardiac dysfunction (CTRCD, n = 50). CTRCD was categorized as symptomatic (very severe, severe, moderate, mild) or asymptomatic (severe, moderate, mild) based on clinical symptoms and/or biomarker changes. Incidence rates are shown with 95% confidence intervals.

*Abbreviations: CTRCD: cancer therapy–related cardiac dysfunction; hs-TnT: high-sensitivity troponin T; NT-proBNP: N-terminal pro–B-type natriuretic peptide.*

**Supplement Table 4: Biomarkers stratified by CTR-CVT patient group**

|  | **total cohort** | **CTR-CVT** | **no CTR-CVT** | **p-value** | **p-value (corr.)** |
| --- | --- | --- | --- | --- | --- |
| Hb [g/dl] | 9.6 (8.8-11.1) | 9.1 (8.5-10.3) | 10.4 (9.3-11.6) | **<0.001** | **0.001** |
| Platelets [x10^9^/l] | 139.5 (80-192) | 115.5 (72.8-192.3) | 157 (106-206.8) | **0.016** | 0.148 |
| Albumine [g/dl] | 37.3 (33.5-39.2) | 35.3 (30.9-38.1) | 38.2 (36.3-40.3) | **<0.001** | **0.004** |
| LDH [U/l] | 195 (166-253) | 208 (170-279) | 182 (146-237) | **0.047** | 0.423 |
| hsTNT [ng/l] (n=77) | 15.3 (9.1-22.2) | 20.2 (11.8-26.2) | 12.7 (8.4-17.2) | **0.003** | **0.025** |
| NT-proBNP [ng/l] (n=98) | 191 (66-502) | 377 (187-950) | 89 (50-193) | **<0.001** | **<0.001** |
| CRP [mg/l] | 11.1 (6.5-39.7) | 23.5 (9.2-43.4) | 8.5 (4.6-18) | **0.002** | **0.015** |
| Ferritin [ng/ml] (n=85) | 542 (221-1127) | 689 (290-1815) | 374 (105-769) | **0.003** | **0.030** |
| IL-6 [ng/l] (n=90) | 9.6 (5.2-26.1) | 17.5 (6.9-33.1) | 6.2 (3.4-14.7) | **0.002** | **0.021** |

Supplement Table 4: Biomarkers measured prior to CAR-T cell therapy, stratified by CTR-CVT patient group. P-values were calculated using the Mann–Whitney U test, and Bonferroni correction was applied for multiple testing.

*Abbreviations: CTR-CVT: cancer therapy–related cardiovascular toxicity; Hb: hemoglobine; LDH: lactat-dehydrogenase; hsTNT: High-sensitivity cardiac troponin T; NT-proBNP: N-terminal pro–B-type natriuretic peptide; CRP: C-Reactive Protein*

**Supplement Table 5: Immune Cell Profiles Prior to Lymphodepletion and Risk of CTR-CVT**

|  | **total cohort** | **CTR-CVT** | **no CTR-CVT** | **OR (95%-CI)** | **p-value** |
| --- | --- | --- | --- | --- | --- |
| CD3+ before lymphodepletion [1/µl] (n=78) | 609 (309-903) | 554 (255-881) | 654 (337-954) | - | 0.322 |
| CD4+ before lymphodepletion [1/µl] (n=78) | 244 (138-454) | 210 (112-323) | 288 (157-511) | - | 0.169 |
| CD8+ before lymphodepletion [1/µl] (n=78) | 251 (165-538) | 276 (154-535) | 237 (180-523) | - | 0.807 |
| CD19+ before lymphodepletion [1/µl] (n=78) | 0 (0-8) | 0 (0-5) | 0 (0-14) | - | 0.637 |
| Blood type (AB0) (n=102) A  B AB  0 | 49  3  4  46 | 26  3  2  20 | 23  0  2  26 | reference category  516x10^6^ (0-∞)  0.84 (0.11-6.7)  0.64 (0.28-1.46) | 0.998 0.873 0.284 |
| Rh- (n=102) | 16 | 10 | 6 | 1.96 (0.63-6.05) | 0.245 |

Supplement Table 3: Immune cell profiles and blood type prior to lymphodepletion, stratified by occurrence of CTR-CVT. P-values are reported without correction for multiple testing, and lymphocyte counts were compared using the Mann–Whitney U test.
